# Supplementary material for: Supramolecular dynamic binary complexes with pH and salt-responsive properties for use in unconventional reservoirs
Source: PLoS One. 2021 Dec 2;16(12):e0260786. doi: 10.1371/journal.pone.0260786 (PMC8638876; doi:10.1371/journal.pone.0260786)
Supplement: S1 Fig — (DOCX) [file pone.0260786.s001.docx]

**Supplementary Information**


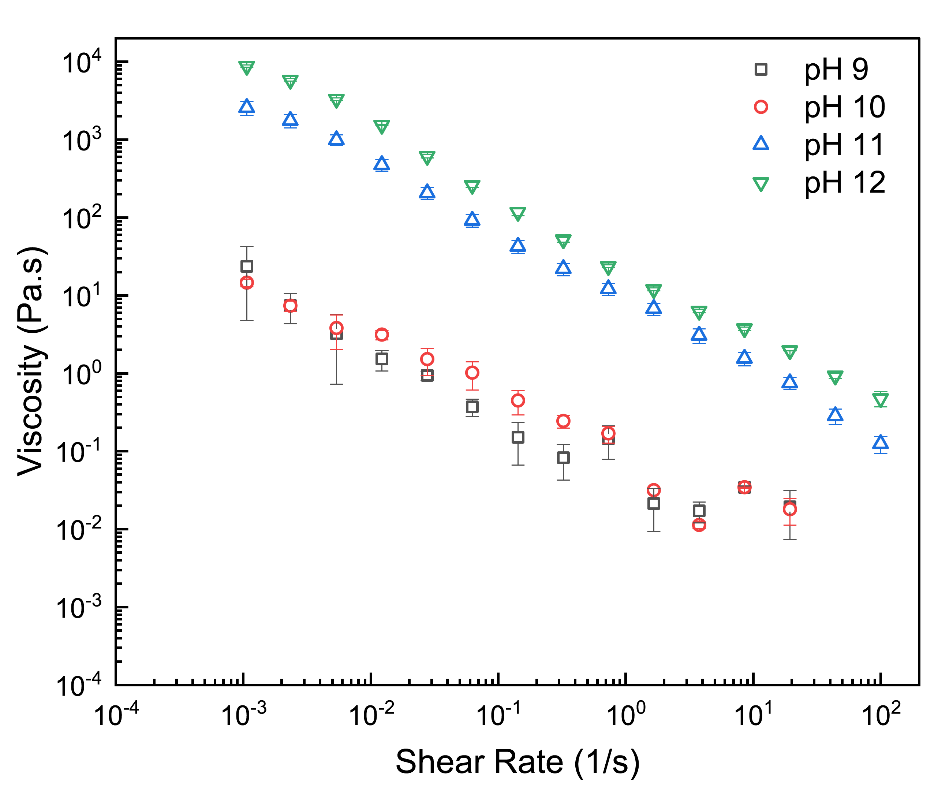


**Fig S1:** Viscosity variation with pH of 2 wt% DBC prepared in simulated sea water
