# Supplementary material for: Supramolecular dynamic binary complexes with pH and salt-responsive properties for use in unconventional reservoirs
Source: PLoS One. 2021 Dec 2;16(12):e0260786. doi: 10.1371/journal.pone.0260786 (PMC8638876; doi:10.1371/journal.pone.0260786)
Supplement: Attachment — Submitted filename: Response Letter v2.docx [file pone.0260786.s002.docx]

Artie McFerrin Department of Chemical Engineering

Texas A&M University

230 Jack E. Brown Engineering Building

College Station, TX 77843, USA

E-mail address: makbulut@tamu.edu

Tel.: +1 (979) 847-8766; Fax: +1 (979) 845-6446

**Mustafa Akbulut, Associate Professor**

Research website: http://research.che.tamu.edu/groups/akbulut

Dear Dr. Scheiber,

We thank the reviewer for judiciously reading our manuscript and for their constructive comments and suggestions on our manuscript, “Supramolecular dynamic binary complexes with pH and salt-responsive properties for use in unconventional reservoirs.” We have carefully considered these comments/suggestions. In light of the feedback we have received, we have revised the article accordingly. Below are our responses to the comments (highlighted in yellow) (Changes are marked with red in the revised main text).

**Editorial Comments:**

The funding information has been removed from the acknowledgements section of the manuscript. We are fine with the current funding statement, and it does not have to be updated. The ORCID ID of the corresponding author has been linked. The reference list was judiciously reviewed, and it was ensured that there were no retracted articles. However, reference 67 (now 70) was noticed to be incomplete and that has been corrected. 3 new references regarding supramolecular systems to adequately capture the state-of-art in this area have been added to the manuscript as well. For Figure 5, we have uploaded a higher resolution image (<10 MB size limit). However, if the 10 MB can be waived, we can upload even higher resolution image that can satisfy the reviewer’s request. Kindly use the high-resolution image if possible.

**Reviewer Comments**

1. Line 88: --> ,

Thank you for pointing out this typographical error. The period in this line has been replaced with a comma, as it is more appropriate.

2. Line 104: in your plots, the minimum shear rate is 10^-4 instead of 0.001 1/s.

We appreciate this comment and see the issue with the axes of the figures. In order to address this, we have changed the figures (Fig1 and Fig2) to ensure that the labelled axes start at a shear rate of 10^-3 (0.001 1/s).

3. Line 110: in cone- plate geometry, it is not possible to adjust the gap. How do you adjust the gap to 1 mm?

Thank you for this observation. We realized that there is an error in this sentence. In fact, we used a 40 mm parallel plate, Peltier plate steel for which gap adjustment is possible. This line has been corrected in the manuscript.

4. Line 110-112: “Dynamic shear frequency sweep measurements were performed on all of the samples at a strain amplitude of 1-10% over an angular frequency range from 0.1 to 100 rad s‒1.” Strain amplitude is constant in oscillatory frequency sweep tests. It is not correct to have a region of 1-10% for strain amplitude. Would you please clarify it?

Thank you for your comments. We meant to say that the strain amplitude chosen was 1% for certain samples (pH 11 and 12) and greater values for the others (4%/10%). This is because a larger amplitude seemed necessary for the instrument to effectively detect the modulus values of less viscous samples. Furthermore, it was possible to have a larger strain amplitude for pH 9 and 10 samples because they have a larger limit of linear viscoelasticity. The above statement has been modified in the manuscript to elucidate this point.

5. Line 119: Why do you choose 7wt% of sand particles for the settling tests? Any calculation according to the real situation? Or randomly was chosen?

Thank you for your question. In literature, proppant settling tests have been conducted with a wide variety of concentration of sand, as low as 5 wt% [1]. Sometimes, the approach has been to just use a single particle of sand and see how fast it settles down after travelling through the fluid [2]. This particular value of 7wt% was chosen as a good starting point which would allow us to gain a visual picture of how well sand is being captured by the matrix of the fluid.

6. Line 143: shear rates unit is 1/s, not HZ!

This has been corrected in the updated manuscript

7. Line 144: increasing the viscosity at a shear rate of 10^-3 s^-1, is not 30-fold by increasing salinity from 0wt% to 3wt%. Please check the amount of viscosity from the plot.

Thank you for this correction. A more accurate statement would be that there is a ~260 fold increase in viscosity at 10^(-3) s^(-1) upon changing the salinity from 0 wt% to 3 wt%. This correction has been made in the main text.

8. Line 223: What is the amount of the imposed shear strain/stress in the angular frequency sweep (fig 4)?

This has been addressed in question 4. The corrected statement in the manuscript provides this information in a better fashion under the ‘Materials and Methods’ section

9. How the error bars are calculated in Fig. 1? Do you repeat the test several times?

Thank you for your question. Each measurement was performed 3 times for statistical analysis. The error bars represent the standard error from the mean of the 3 measurements. We recognize this has not been mentioned clearly in the text. Hence, we have added an explanation in the materials and methods section.

10. Could you provide higher resolution images in Fig 5?

Due to the image size limit, Figure 5 had to be compressed. Some resolution was lost because of this. A slightly better version that is under 10 MB has been uploaded. The link for the original high resolution file has been shared with the PLOS One as well.

1.	Ahmad FA, Miskimins JL. Proppant Transport and Behavior in Horizontal Wellbores Using Low Viscosity Fluids. 2019. doi:10.2118/194379-MS

2.	Shah SN. Proppant Settling Correlations for Non-Newtonian Fluids Under Static and Dynamic Conditions. Soc Pet Eng J. 1982;22: 164–170. doi:10.2118/9330-PA

Sincerely,

**Mustafa Akbulut, Ph.D.**
